# ‘SAXS-osmometer’ method provides measurement of DNA pressure in viral capsids and delivers an empirical equation of state

**DOI:** 10.1093/nar/gkad852

**Published:** 2023-10-27

**Authors:** José Ramón Villanueva Valencia, Dong Li, Sherwood R Casjens, Alex Evilevitch

**Affiliations:** Department of Experimental Medical Science and NanoLund, Lund University, Box 124, Lund, Sweden; Physics Department, Carnegie Mellon University, Pittsburgh, PA 15213, USA; Division of Microbiology and Immunology, Department of Pathology, University of Utah School of Medicine, Salt Lake City, UT 84112, USA; Department of Experimental Medical Science and NanoLund, Lund University, Box 124, Lund, Sweden; Physics Department, Carnegie Mellon University, Pittsburgh, PA 15213, USA

## Abstract

We present a novel method that provides a measurement of DNA pressure in viral capsids using small angle X-ray scattering (SAXS). This method, unlike our previous assay, does not require triggering genome release with a viral receptor. Thus, it can be used to determine the existence of a pressurized genome state in a wide range of virus systems, even if the receptor is not known, leading to a better understanding of the processes of viral genome uncoating and encapsidation in the course of infection. Furthermore, by measuring DNA pressure for a collection of bacteriophages with varying DNA packing densities, we derived an empirical equation of state (EOS) that accurately predicts the relation between the capsid pressure and the packaged DNA density and includes the contribution of both DNA–DNA interaction energy and DNA bending stress to the total DNA pressure. We believe that our SAXS-osmometer method and the EOS, combined, provide the necessary tools to investigate physico-chemical properties of confined DNA condensates and mechanisms of infection, and may also provide essential data for the design of viral vectors in gene therapy applications and development of antivirals that target the pressurized genome state.

## Graphical Abstract

**Graphical Abstract:**
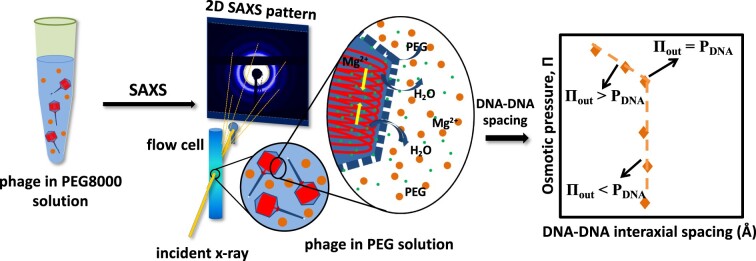

## Introduction

The mechanisms by which viruses deliver their capsid-protected genomes into host cells are poorly understood. One of the main viral strategies for genome uncoating is pressure-driven viral DNA ejection from an intact capsid into a host, based on the discovery of tens of atmospheres of DNA pressure inside capsids of double-stranded (ds) DNA bacteriophages (1,2) (throughout the text, we use dsDNA and DNA terms interchangeably), archaeal viruses (3) and herpesviruses (4); other uncoating strategies involve capsid disassembly, directional genome uncoating as part of capsid functionality and others (5). The evolutionary conservation of a pressure-driven DNA release mechanism across virus families, which infect cells from all three domains of life, suggests it is one of the central traits of the viral infectious cycle. Intracapsid genome pressure is generated by packaging a micrometer-long dsDNA into a nanometer-size capsid by an ATP-driven motor complex on the capsid, which is the strongest molecular motor known (6). This tight packaging results in repulsive electrostatic forces between negatively charged neighboring DNA helices and bending stress on the packaged genome due to the stiffness of dsDNA (7,8). Quantification of the pressurized state of an encapsidated viral genome is required for understanding key mechanistic steps of the infectious cycle, e.g. genome packaging during viral assembly and its subsequent ejection into a cell. Furthermore, knowing whether a virus is in a pressurized intracapsid genome state is important for development of new antiviral treatments (9) and biomedical technologies. For example, we recently provided a proof-of-concept for an antiviral mechanism of action (MOA) targeting the pressurized state of DNA in herpesvirus capsids by small molecule polycationic compounds. These specific compounds were shown to penetrate the viral capsid and ‘turn off’ the DNA pressure by condensing the genome, which subsequently blocked DNA ejection into a cell, preventing herpesvirus replication. This MOA does not lead to development of drug resistance, unlike most antiviral drugs targeting viral proteins, which are prone to rapid mutations (9). A similar antiviral approach could be applied to other viruses, if a pressurized intracapsid genome state can be identified.

Yet, the intracapsid genome pressure has only been measured for a handful of viruses over the past decades (1,3,4,10,11). [Here and throughout the text, we refer to viruses with a positive outward genome pressure on the capsid walls, which occurs when genome is packaged into a preformed capsid under stress. It should be mentioned that there are other virus systems where positively charged capsid subunits co-assemble with the negatively charged genome, which results in an attractive interaction and a small negative genome pressure pulling the capsid together (12).] The major limiting factor preventing a systematic screening of virus systems for pressurized genome state lies in the pressure measurement method. For example, the ‘osmotic suppression assay’ (1), which we originally designed, requires isolation of a virus receptor from cells (1); the solubilized viral receptor is added to a solution with purified viral particles, *in vitro*, which triggers complete genome ejection from viral capsids into the surrounding solution. The extent of ejected viral genome length is progressively suppressed by addition of an external inert osmolyte, e.g. PEG 8000 (MW ≈ 8000 Da) at increasing concentration. Since PEG does not penetrate the pores on viral capsid (all viral capsids are permeable to water and smaller ions but not to larger polymers), this generates an osmotic pressure gradient between the exterior and interior of the capsid, resulting in an osmotic force suppressing the DNA ejection. Next, the external osmotic pressure is recorded as a function of DNA length remaining suppressed in the capsid after receptor treatment. When external osmotic pressure is equal to that of DNA pressure in the capsid, no DNA ejection occurs, which determines DNA capsid pressure(1). With this approach, we demonstrated and quantified DNA pressure in bacteriophage λ (*P*_DNA_∼35 atm) (1) and later in a eukaryotic virus, human herpes simplex virus type 1 (HSV-1) (*P*_DNA_∼20 atm) (4). However, for most viruses, the viral receptors required for this assay are unknown or cannot be successfully isolated (without losing the ability to trigger genome release). Furthermore, even when a viral genome can be ejected *in vitro* through addition of an isolated receptor, the genome ejection process can significantly differ between different virus types. For instance, DNA ejection from phage P22 (10) does not exhibit a direct correlation between the external osmotic pressure and the remaining DNA length suppressed in the capsid, as found for phage λ (DNA ejection from P22 contains ‘ejection proteins’ in the capsid that are released upon receptor addition preceding DNA ejection, resulting in an instant DNA pressure drop unrelated to the ejected DNA length). This precludes a direct comparison of capsid pressures between different viruses. In this work we present a novel method that allows accurate determination of positive genome pressure in viral capsids that can likely be applied to a wide range of different viruses, without needing a viral receptor to trigger viral genome ejection *in vitro*. The DNA pressure is determined directly in a fully packaged viral capsid. Hence, the mechanism of genome ejection does not influence the measurement. The method is based on correlation of an increasing external osmotic pressure, generated by PEG, with DNA–DNA spacing, *d_s_*, in fully packaged intact viral capsids, measured by small-angle X-ray scattering (SAXS). The physico-chemical principles governing this method are outlined in the next section. This provides a universal, label-free pressure screening assay, allowing discovery and standardizing comparison of internal capsid pressures between different viruses.

Furthermore, we determined how internal DNA capsid pressure varies with the DNA packaging density measured for a collection of different bacteriophages (for several of which the receptors are unknown). Using this data, we derived a semi-empirical equation of state (EOS) for encapsidated DNA. Most of the cellular building blocks—DNA, proteins and lipids are subjected to mandatory condensation inside a cell, due to molecular crowding (13,14). Specifically, intracellular DNA (both cell chromosome and viral DNA) is condensed by direct specific or non-specific interactions with DNA-binding molecules (e.g. DNA-binding proteins and polyamines) acting in synergy with molecular crowding (15). Knowledge of physico-chemical properties of condensed biomolecular phases is therefore critical for understanding of molecular interactions controlling intra- and intermolecular intracellular mechanisms. An EOS, carries this information by providing a relationship between the state variables, such as osmotic pressure and solute (e.g. DNA) concentration, under a given set of physico-chemical conditions. A semi-empirical EOS relation has been previously derived for free linear dsDNA arrays condensed in a bulk solution by an osmolyte (16). This work was seminal for discovery of a new physical force, a repulsive hydration interaction between densely packed biomolecules. The hydration force dominates the interactions between densely packed DNA-strands over the electrostatic force, at center-to-center separations (*d_s_*) of ∼20–30 Å (3). This DNA packaging density is also typical for DNA packaged in viral capsids (17). However, the principal difference between linear DNA arrays condensed in bulk and DNA confined inside a capsid is the additional bending stress imposed on the packaged DNA by the curvature of the spherical capsid walls (7,18,19). Until now, the relative contribution of the bending energy term to the total DNA pressure in capsids remained unknown, with no available data allowing such quantification. Our empirically derived EOS describes the thermodynamic properties of encapsidated DNA and includes contribution to DNA pressure from both DNA–DNA interaction energy and bending energy terms. Besides prediction of pressure in viral capsids, EOS for intracapsid DNA explains how different parameters (packaged genome length, capsid diameter or external solution conditions) regulate the internal genome stress, which can facilitate or inhibit genome uncoating and packaging (9,20). This information is indispensable for optimization of packaging conditions for efficient encapsidation of oversized viral vectors in viral gene therapy applications (21) and other biotechnologies.

## Materials and methods

### Phage purification

WT bacteriophage λ cI857, with a genome length of 48.5 kb, was produced by thermal induction of lysogenic *Escherichia coli* strain AE1 derived from S2773 strain. Phage purification details were described elsewhere (22). A shorter genome length phage λ mutant (78% of the WT DNA length) was produced using a similar procedure. All phage samples were purified by CsCl equilibrium centrifugation and dialyzed from CsCl against MgSO_4_ TM buffer (10 mM MgSO_4_, 50 mM Tris·HCl, pH 7.4). Bacteriophages P22, 9NA, and Utah were kindly provided by Eddie Gilcrease and Sherwood Casjens at University of Utah School of Medicine.

### X-ray measurement of DNA–DNA forces in condensed arrays

Force measurements were carried out at the Laboratory of Physical and Structural Biology, Program in Physical Biology, National Institutes of Health. Ni-filtered Cu-Ka radiation from an UltraBright microfocus x-ray source from Oxford Instruments equipped with polycapillary focusing X-ray optics was used for the small angle X-ray scattering (SAXS) experiments. The primary beam was also collimated by a set of slits. The flight path between the sample and detector, ∼ 16 cm, was helium filled. Typical exposure times were ∼ 30 min. Calf thymus DNA was ordered from Sigma. A typical phenol/chloroform-chloroform extraction procedure was used to purify the DNA, which was then EtOH precipitated and dried at room temperature. For each experiment, about 200 μg DNA was washed with EtOH and followed by a brief spin. Then the DNA pellet was washed in 70% EtOH followed by a spin. The pellet was transferred to ∼ 1 ml of polyethylene glycol (PEG8000) solution. The bathing PEG solution was changed once or twice after several days. After equilibration, samples were sealed with equilibrating PEG solution in a sample cell and mounted into a temperature-controlled holder. At equilibrium, the osmotic pressures in both PEG and DNA phases are equal. The interaxial spacing of condensed DNA arrays was determined by X-ray scattering as a function of the applied PEG stress. Further details are described elsewhere (23).

### ‘SAXS-osmometer’ measurements of DNA pressure in viral capsids

To determine DNA pressure in phage, PEG 8000 was added to phage solutions in Tris–MgSO_4_ (TM-buffer) at 37°C, at progressively increasing PEG concentrations from 0 to 45% by weight (w/w) corresponding to a maximum osmotic pressure of ∼65 atm. Due to difficulty of pipetting the viscous PEG solutions, we tried to minimize systematic and statistical errors that occur when the solution adheres to the pipette tips. A stock solution of 50% by weight (w/w) PEG 8000 was prepared in Tris-MgSO_4_ (TM-buffer). This stock solution was used to prepare solutions of PEG/TM-buffer at various specified % (w/w) on an analytical balance within an error in concentration of approximately ∼0.1% (w/w). Each sample was incubated for 1 hour in the corresponding PEG solution, allowing equilibration of the intracapsid DNA state, which was verified in ref(1). DNA–DNA spacing, *d_s_*, was then measured with SAXS for DNA packaged in phage capsids as a function of increasing PEG concentration. This was achieved by mixing stock solution of phage (at concentration ∼10^13^ pfu/ml) with 50% w/w of PEG8000 stock solution. Other details and method description are provided in the main text.

### Small-angle X-ray scattering:

Small-angle X-ray scattering (SAXS) measurements were carried out at the 12-ID B station at the Advanced Photon Source at Argonne National Laboratory. A 12-KeV X-ray beam was used to illuminate the sample with an overall scattering vector *q* range from 0.0025 to 0.52 Å^−1^. For non-PEG samples, a total of 120 μl of phage solution (∼1 × 10^13^ pfu/ml) was injected into a flow-through glass capillary, and the solution was oscillated during the SAXS measurement with a flow rate of 10 μl/s. Forty scans with 1-s X-ray exposure time were collected and averaged for each sample. When measuring phage samples with PEG8000, the viscous solution was slowly injected into the glass capillary. The sample was allowed to incubate at 37°C for 15 min prior to the SAXS measurements. 5–10 scans were collected and averaged without oscillating the sample. After the background subtraction, the scattered intensity I versus q was plotted and the DNA peak region was truncated from 0.16 Å^−1^ to 0.35 Å^−1^. The DNA diffraction peak was then fitted with a broad peak model using the function below, where *q*_0_ is the peak center, *A* is the Porod law scale factor, *n* is the Porod exponent, *C* is the Lorentzian scale factor and ξ the screening lenght. From the peak center, the DNA–DNA interaxial spacing *d*_int_ was calculated as ${d}_{int}{\mathrm{\ }} = 4{\mathrm{\pi }}/\sqrt 3 {q}_0$, assuming hexagonal close packing of DNA arrays inside the phage capsid.


\begin{equation*}I = \frac{A}{{{q}^n}} + \frac{C}{{1 + {{\left( {\left| {q - {q}_0} \right|\varepsilon } \right)}}^2}}\end{equation*}

## Results and discussion

First, in ‘*Using SAXS as an osmometer’* we outline the physical principles of the new method, designed to measure DNA pressure in viral capsids without triggering genome ejection with a viral receptor. Next, as a proof-of-concept, we measure DNA pressure in phage λ capsids packaged with 100% wt-DNA and with 78% of wt-DNA length using our new approach, providing a direct comparison with the previously obtained DNA pressure values in these phage systems using the assay requiring DNA ejection with an isolated λ-receptor (1). This data is presented in *‘Measuring intracapsid genome pressure without DNA ejection’*. Finally, we derive *‘The Equation of State for encapsidated DNA’* by expanding DNA pressure measurements to different phages with varying DNA packaging densities (thus, variable *d_s_*-values). DNA pressures versus *d_s_* are plotted on a semi-log scale and a linear equation of state (EOS) is derived, which includes both DNA–DNA interaction and bending energy terms. Our semi-empirical EOS and the new pressure measurement method combine to provide a measurement of DNA bending stress contribution to the total capsid DNA pressure in different viruses. The new SAXS-osmometer method can be applied to a wide range of viruses with a positive genome pressure inside the capsid and will advance our knowledge of processes of viral genome packaging and release. [Our method cannot be extended to virus systems with negative genome pressure, where capsid proteins and viral genome co-assemble under attractive interactions.]

### Using SAXS as an osmometer

Consider a situation when DNA-filled viral capsids are in an aqueous buffer solution without osmolite present. Due to high DNA concentration inside the capsid, water is drawn into the fixed capsid volume (due to entropic drive to maximize mixing) and a large hydrostatic pressure is developed inside the capsid to equalize the chemical potential of the water throughout the system (in the ‘‘inside’’ and ‘‘outside’’ solutions). This hydrostatic pressure, due to the water pressurized inside the rigid volume (and withstood by the rigid walls of the protein capsid), is what we describe as DNA intracapsid pressure, *P_DNA_* (24). For pressurized viruses, dsDNA packaged inside the capsid is ordered in a hexagonal lattice structure (25,26). As mentioned above, DNA–DNA interaction and DNA bending are two main energy terms contributing to the DNA pressure and determining the spacing, *d_s_*, between the packaged DNA strands (7,27). To reduce the bending energy, DNA will be pushed towards the capsid walls (providing the lowest curvature), whereas repulsive DNA–DNA hydration interactions will push the DNA strands as far from each other as possible, filling the capsid volume and maximizing the interstrand surface separation (24). The balance between these two energies determines the value of *d_s_* (7,27). [The tight DNA packaging into a capsid results in dehydration of the DNA backbone. It has been shown that, for hexagonally ordered DNA, the hydration entropy dominates over the loss of conformational entropy of the DNA coil upon confinement in the capsid and has the opposite sign (28,29). The entropic part of DNA–DNA hydration interaction energy will be discussed below]. It follows that a fixed concentration of DNA inside the rigid capsid walls corresponds to a particular fixed value of interaxial spacing between DNA strands. Suppose we now add PEG to the outside solution (with PEG polymer size larger than the capsid pores so it cannot penetrate the capsid). Water will be drawn out of the DNA volume, reducing the water density and the hydrostatic pressure inside. Let c*_PEG_ be the concentration of PEG that brings the pressure inside the capsid down to 1 atm (atmospheric pressure). For this special value of *c_PEG_*, water-exchange equilibrium corresponds to zero osmotic (hydrostatic) pressure difference and a net force of zero on the rigid walls confining the DNA. Equivalently, the osmotic pressure, *Π*, associated with the concentration c*_PEG_, is equal to the DNA osmotic pressure, *P*_DNA_, exerted by the confined DNA with spacing *d_s_*. As a consequence, even if the DNA was allowed the opportunity to ‘‘escape’’ from its confinement, it would not because there is no thermodynamic driving force for this process (24). Therefore, in our previous osmotic suppression assay measuring DNA pressure in phage λ(1), when the capsid portal was opened with solubilized LamB maltoporin receptor in the presence of PEG 8000 with concentration c*_PEG_, no DNA was ejected (corresponding to an osmotic pressure of ∼25–35 atm equal to DNA pressure in the capsid (30).

In our new method determining the intracapsid pressure in a closed DNA-filled capsid, for any lower external PEG concentration value of *c*_PEG_ than c*_PEG_, there is a pressure difference and hence a net force (outward) on the confining capsid walls, because an insufficient amount of water has been drawn out of the DNA solution to lower its hydrostatic pressure to 1 atm. Therefore, DNA–DNA spacing in the capsid should remain constant for all *c*_PEG_ ≤ c*_PEG_, when DNA bending and DNA–DNA repulsion are not balanced by the PEG osmotic pressure. However, if PEG concentration is increased further to *c*_PEG_ > c*_PEG_ this will create an under-pressure on DNA in the capsid (as water hydrating DNA strands will be removed from the capsid to instead hydrate the more concentrated PEG solution outside the capsid walls). As a result, DNA will start condensing inside the capsid, resulting in an interaxial spacing decreasing below that of the initial d*_s_-value. [Here, we use the term ‘condensation’ to refer to DNA compaction induced by PEG, defined as Ψ-condensation (31). PEG is a biologically inert polymer and induces DNA condensation through depletion forces.] It was previously demonstrated that SAXS measurements of pressurized DNA-filled viral capsids allow precise determination of the average interaxial spacings *d_s_*-value between ordered DNA strands packaged inside viral capsid, reflected by a Bragg peak for hexagonally ordered DNA lattice (32,33). Thus, by using SAXS to determine *d_s_*-values for intracapsid DNA versus increasing PEG 8000 concentration, we can find PEG concentration c*_PEG_ when *d_s_*-value starts to decrease. At d*_s_-value, the intracapsid DNA pressure is equal to the external osmotic pressure. Thus, the SAXS-measured Bragg peak from packaged DNA structure is used as an osmometer (23) to measure internal capsid pressure *P*_DNA_, without a requirement of ejecting the DNA with a receptor. [Our previous cryo-EM data (19) shows that DNA packaged in phage λ capsid displays a hexagonally ordered DNA phase in the periphery of the capsid (occupying most of the capsid's volume) and a disordered DNA phase with a lower density in the core of the capsid. These two coexisting phases allow variation in *d_s_*-spacing in the ordered DNA phase while the λ-DNA molecule occupies the entire capsid volume (in order to maximize the DNA–DNA separation and minimize the DNA bending stress). With increasing PEG concentration, when *d_s_* is no longer constant (at *c*_PEG_ > c*_PEG_) and DNA does not exert a net pressure on the capsid walls, λ-DNA could either continue to occupy the entire capsid volume (so that the λ-DNA amount partitioned between the core and the periphery intracapsid phases is shifted) or become further condensed, occupying less volume than available in the capsid. Neither of these scenarios affect our capsid pressure determination since capsid pressure and EOS are derived at c*_PEG_ with DNA occupying all available capsid volume, as explained below.]

It is important to note here that pressure exerted by confinement of the tightly packaged, negatively charged DNA chain inside the capsid and pressure exerted by water hydrating the packaged DNA describe one and the same phenomena, referred to as capsid genome pressure. It has been shown that, as DNA is being progressively packaged into a capsid by the capsid portal motor, the force and pressure on the capsid walls are continuously building up (34). As described above, the force is generated by DNA bending stress induced by a confined capsid volume and by DNA–DNA interaction energy. Parsegian *et al.* (23) showed that the net repulsive interaction between negatively charged DNA helices, as they are being packaged, is initially predominantly electrostatic (there is also a van der Waals attraction term). However, as the DNA packaging proceeds and interhelical distance reaches that of <∼30Å, the repulsive interaction is dominated by hydration forces, which are independent of the ionic conditions in the solvent media bathing the capsid. Thus, for a partially DNA-filled capsid, the genome pressure is dictated by DNA–DNA electrostatic repulsive forces and bending stress (thus, the DNA chain itself), while for a fully DNA-packaged phage capsid (as in our study), the capsid pressure is determined by the interstrand hydration force (from water permeating the capsid to hydrate the DNA) and the bending stress. We have previously confirmed this assumption with a stringent test (35). In this experiment, Herpes Simplex virus (HSV-1) capsids, which have high DNA capsid pressure(20 atm) similar to that of phage λ, were adsorbed on the surface of isolated cell nuclei; upon binding to the nuclear pore complexes (NPCs), capsids are shown to instantly eject their DNA into the host nuclei *in vitro*, since NPCs ‘unplug’ the capsid portal letting the DNA out (35). When PEG8000 was added to the surrounding solution to generate an osmotic pressure matching that of capsid pressure, no ejection occurred. In this experiment, PEG is present outside the capsid and outside the nuclei (its molecular weight does not permit permeation of either). Thus, it only affects the chemical potential of water, which can freely permeate the capsid. As water hydrating the DNA (and generating the pressure) is partially removed through the capsid pores by PEG addition, there is no excess pressure left in the capsid and DNA ejection is blocked. The change in the conformational entropy of DNA ejection does not need to be taken into account since entropy term is dominated by DNA hydration entropy (28), as will be explained below.

In the section below, as a proof-of-concept, we demonstrate the robustness and reproducibility of this method by first measuring DNA pressure in phage λ with two packaged DNA lengths, corresponding to wild-type (wt) λ-DNA of 48500 bp and its shorter λ-DNA length mutant of 37 800 bp (corresponding to 78% of the wt λ-DNA length). We selected these two phage systems since obtained DNA pressures can be directly compared to our previously obtained pressure values using the osmotic suppression assay (1).

### Measuring intracapsid genome pressure without DNA ejection

**Figure 1.**
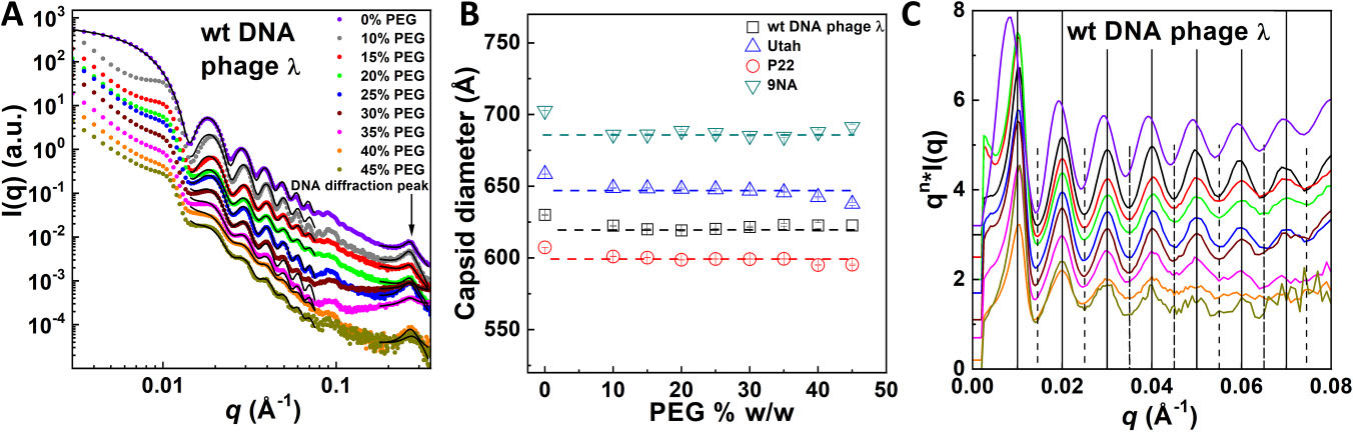
(**A**) SAXS scattering intensity profiles, *I*, versus magnitude of the scattering vector *q* for wt DNA phage λ in Tris-MgSO_4_ buffer at 37ºC with PEG8000 added between 0 and 45% w/w. The black solid lines in the low to middle *q*-region are the spherical model fit used to determine capsid diameter for each *I(q)* curve. The solid black lines in the high *q*-region show the broad-peak model fit to locate the DNA diffraction peak position reflecting hexagonally ordered DNA structure in the capsid. (**B**) Outer capsid diameter for phages wt λ, Utah, P22 and 9NA as a function of PEG 8000 concentration determined by fitting the SAXS intensity patterns in Figure 1A with a spherical model. Data demonstrates that capsid size does not change with PEG concentration increment. (**C**) Porod–Debye-like plot, q^n^*I(q) versus *q*, with *n*= 3.4 for all SAXS profiles for wt phage λ (showing only the low to middle *q*-regions data from Figure 1A). SAXS curves are vertically offset for clarity and vertical lines are drawn to show positions of the valleys/peaks.

As described above, DNA–DNA spacing, *d_s_*, was measured with SAXS for DNA packaged in wt phage λ and 78% DNA phage λ, respectively. PEG 8000 was added to phage λ solutions in Tris–MgSO_4_ (TM-buffer) at 37°C, at progressively increasing PEG concentrations from 0 to 45% by weight (w/w) corresponding to a maximum osmotic pressure of ∼65 atm. Prior to the SAXS measurement, each sample was bathed in the corresponding PEG solution for 1 h, which ensures equilibration of the intracapsid DNA state (1). PEG 8000 concentration was converted to osmotic pressure using the empirical relation *Π*(atm) = –1.29 *G^2^T + 140G^2^ + 4G*, where *G = w*/(100 – *w*) with *w* being the weight percent (% w/w) PEG and *T* is the temperature in the range 5°C < *T* < 40°C (23). Solution SAXS provides structural information about both capsid and encapsidated DNA structures. First, we confirmed that capsid size and shape were not influenced by the high osmotic pressure gradient experienced by the exterior of the capsids, which would otherwise affect the measured *d_s_*-values. Figure 1A shows SAXS scattering intensity profiles, *I*, versus magnitude of the scattering vector *q* (called wave number) for wt phage λ at all PEG concentrations (similar data for 78% DNA phage λ are shown in Figure S1 in the Supplemental Materials). Background solvent scattering *I*(*q*) was measured separately for TM-buffer at 37°C and subtracted from all scattering profiles. The interpretation of the structure reflected by the scattering intensity pattern depends on the value of the wave number *q*, which is inversely proportional to the length scale *L* of the analyzed structure, i.e. $q\sim{L}^{ - 1}$. Thus, the scattering signal in the low to middle *q*-region ∼ 0.006–0.07 Å^−1^ provides scattering information at the large-length scales (tens to hundreds of nm), corresponding to the whole viral particle and reflecting structures of both capsid and global packaged DNA arrangement inside (called the ‘form factor’ in the scattering analysis). On the contrary, in high *q*-region ≥0.1 Å^−1^, Bragg peak reflects the coherent scattering at short-length scales (tens of Angstroms) from the averaged local ordering of dsDNA helices packaged in a capsid (called the DNA ‘structure factor’ in the scattering analysis), providing average distance between ordered DNA centers, *d_s_* (32,33). Thus, SAXS data for phage λ capsid can be interpreted by fitting the scattering intensity versus *q*. We use two fitting models that separately describe the data at low-middle *q*-region and high *q*-region (black lines in Figure 1A). At low-middle *q*, a simple spherical model (approximating the icosahedral capsid with a sphere) describes the scattering profile (∼0.01–0.075 Å^−1^), providing information on the capsid size (while capsids have a faceted icosahedral structure, the spherical model is sufficient for the purpose of validating capsid size invariance with addition of PEG). Figure 1A shows a good agreement between the SAXS data and the fitting model. The model fit yields the spherically averaged capsid diameter for λ-capsids of ∼63 nm (see Table 1), in agreement with our previous cryo-EM data (36). Capsid diameter remains constant at all PEG concentrations, Figure 1B.

**Table 1.** Capsid size, packaged DNA length, interstrand distance, and measured capsid DNA pressure for the collection of phages used in this study

**Phage type**	**Capsid diameter (nm)**	Inner capsid diameter (nm)*	**DNA length (kbp)**	**DNA–DNA spacing (Å)**	**DNA pressure (atm)**
P22	63^[1,2]^	56.7	43.5	26.8 ± 0.1	39 ± 9
wt DNA phage λ	63^[3,4,5]^	59.7	48.5	27.6 ± 0.2	34 ± 5
78% DNA phage λ	63^[3,4,5]^	59.7	37.8	29.9 ± 0.1	15 ± 6
9NA	68^[6]^	64.2	59	27.9 ± 0.1	29 ± 8
Utah	67^[7]^	62.4	59	26.7 ± 0.2	37 ± 10

^1^J Tang *et al.*, Structure **19** (4), 496 (2011).

^2^Y Jin *et al.*, Virology **485**, 128 (2015).

^3^T Dokland and H Murialdo, J Mol Biol **233** (4), 682 (1993).

^4^GC Lander *et al.*, Structure **16** (9), 1399 (2008).

^5^GC Lander *et al.*, Nucleic Acids Res **41** (8), 4518 (2013).

^6^C Zeng *et al.*, J Virol **93** (22) (2019).

^7^JC Leavitt *et al.*, Genome Announc **5** (13) (2017).

*Inner capsid diameter is estimated using SAXS data at 0% PEG.

It can be noted, that with addition of PEG, we observed the appearance of an additional broad peak and flattening in the low *q*-region of SAXS scattering profiles (*q* ∼ 0.009–0.01 Å^−1^) at all PEG concentrations. Furthermore, the observed plateau in *I*(*q*) in the low *q*-region (<0.008 Å^−1^) when *q* → 0 (in the log-log plot), without PEG added, is replaced with an increasing *I*(*q*) function as *q* → 0 with PEG, see Figure 1A. These changes indicate known formation of partial capsid aggregates induced by PEG addition, which are not large enough to cause macroscopic precipitation. The aggregate formation is reflected by the appearance of the capsid-capsid structure factor (the observed extra peak in the low *q*-region). The position of the structure factor peak at low *q*, however, remains unchanged at all PEG concentrations between 10% and 45% w/w. To further analyze the effect of PEG addition on the capsid size and shape, in Figure 1C, we show a Porod-Debye-like plot, *q*^n^*I(*q*) versus *q*, with *n* = 3.4 for all SAXS profiles for wt phage λ (showing only the low *q*-region data from Figure 1A). The positions of the valleys and peaks in the *q* range up to 0.1 Å^−1^ are sensitive to the change in the particle size and shape, where even a 1 Å change in particle radius results in a significant shift of the valley/peak positions. SAXS curves are vertically offset for clarity and vertical lines are drawn to show positions of the valleys/peaks. The positions of all valleys/peaks in the SAXS profiles for samples with added PEG were only slightly, synchronously shifted toward higher *q*-values compared to the zero-PEG sample, see Figure 1C. However, the small shift was identical at all PEG concentrations between 10 and 45% w/w and periodicity of the valleys/peaks was unaffected by PEG addition at all PEG concentrations, demonstrating that capsid size and shape are unaffected by high osmotic pressures. The observed position shift occurs due to the fact that scattering length density (SLD) of the whole solution (including both viral particles and the surrounding PEG buffer) is higher than SLD in the zero-PEG buffer and closer to that of the protein capsid. Therefore, SAXS scattering signal contribution of the protein capsid shell becomes reduced in the presence of PEG, and apparent particle size appears slightly smaller. This is observed from the capsid dimeter values obtained from the spherical model fit of SAXS data, shown in Figure 1B. In addition, as mentioned above, PEG-induced formation of capsid aggregates promotes the appearance of the capsid–capsid structure factor at low-*q*'s, which can also cause a small shift of the consecutive valleys/peaks. Since PEG-induced external osmotic pressure gradient does not lead to capsid deformation, with capsid volume remaining constant, we could proceed with the SAXS analysis of DNA–DNA interaxial spacing of packaged DNA as a function of increasing osmotic pressure, in order to determine the DNA pressure in the capsid.

**Figure 2.**
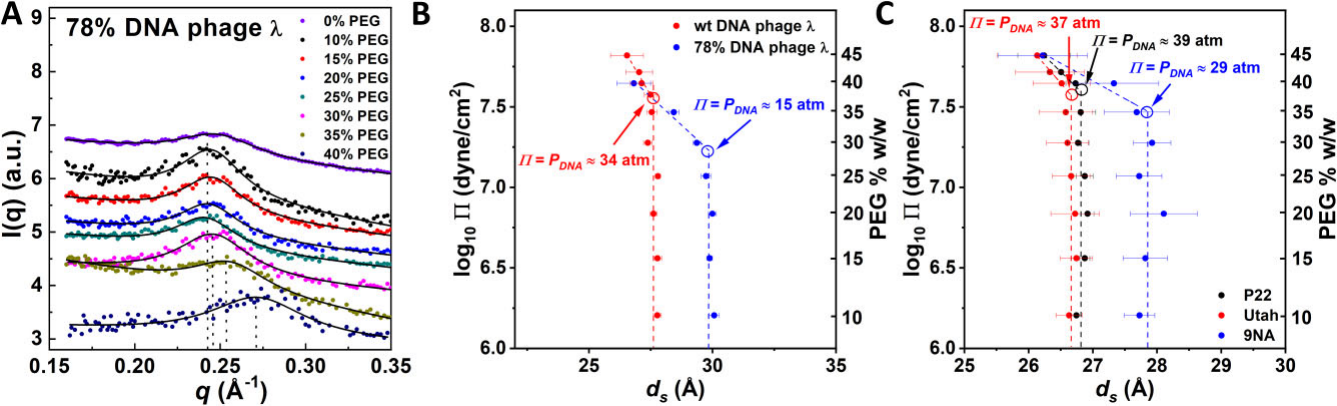
(**A**) SAXS-obtained DNA diffraction peak intensity profile, *I*, versus *q* (reflecting the DNA structure factor) in the high *q*-region (small length scales) at different PEG concentrations for 78% of wt DNA length phage λ. The broad-peak model (black lines) is used to fit the DNA peak to determine the peak maximum value. (**B**) Osmotic pressure plotted as log_10_*Π* versus *d_s_*-values for DNA packaged in wt phage λ and 78% DNA phage λ. Data shows that addition of PEG below the critical concentration *c*_PEG_*, where *P_DNA_* (DNA pressure in the capsid) ≤ *Π* (osmotic pressure), is insufficient to further condense DNA in the capsid, hence *d_s_*-values remain constant. However, when PEG concentration reaches *c*_PEG_* and the osmotic pressure in solution exceeded that of DNA pressure in the capsid (*c*_PEG_ > *c*_PEG_* corresponding to *Π* > *P*_DNA_), further DNA condensation occurs, with *d_s_* gradually decreasing as PEG concentration is increasing. (**C**) log_10_*Π* versus *d_s_* for each of the phages P22, Utah and 9NA. At *c*_PEG_≤ c*_PEG_, *d_s_-*values for intracapsid DNA remained constant for all phages. At *c*_PEG_> c*_PEG_, *d_s_-*values showed a linear decrease with increasing osmotic pressure on semi log-scale. The intercept between the linear fit for d_s_< d_s_* and the vertical line at constant d_s_* provides the DNA pressure value in the capsid, where PEG osmotic pressure, *Π*, in solution is equal to intracapsid DNA pressure, *P_DNA_*. All phages are in 10 mM Tris–MgSO_4_ buffer at 37ºC. The horizonal error bars in B and C are one standard deviation, σ_q0_, where *q*_0_ is the peak center for the location of the Bragg's peak maximum for ordered DNA in phage determined with the ‘broad peak’ model. The obtained uncertainty in the osmotic pressure values (*Π*) varies with PEG concentration and is between ∼1% and ∼5% atm. On the semi-log representation of *log_10_Π* versus *d_s_* in Figure 2, however, these vertical error bars are smaller than the symbols shown in the figure.

As described above, at high *q* (0.15–0.35 Å^−1^), the broad peak model is used to fit the observed Bragg peak (reflecting the hexagonally ordered lattice of DNA packaged in the capsid), which can then be used to accurately locate the position of DNA peak maximum that reflects the interaxial distance between ordered DNA strands in the capsid (37,38). The broad peak model consists of a power law term (usually referred in other contributions to as linear background) and a Lorentzian term (39,40). Previous X-ray scattering studies of bulk DNA phases condensed by an osmolyte (16), have demonstrated that at interaxial DNA–DNA distances of < ∼30 Å, DNA displays a long-range ordering structure with a hexagonal lattice (41), consistent with the DNA structure in phage λ, packaged within the same density range (19,32,42). Figure 2A shows the characteristic Bragg peak for hexagonally ordered DNA in 78% DNA phage λ (i.e. 78% of the wt λ-DNA length of 48 500 bp) in TM-buffer at 37°C without and with PEG8000 addition with an increasing PEG concentration from 0 to 40% w/w. After determining the Bragg peak position (*q_Bragg_*) with the broad peak model (black line fit in Figure 2A), we obtained *d_s_-*values using the relationship for a hexagonal lattice ${d}_s = \frac{{4\pi }}{{\sqrt 3 {q}_{Bragg}}}$. In Figure 2B we converted PEG concentration to osmotic pressure, *Π*, and plotted log_10_*Π* as a function of *d_s_*-values for DNA packaged in wt phage λ and 78% DNA phage λ (see raw SAXS data in Figure S1 in the Supplemental Materials). As explained above, we expect that addition of PEG below the critical concentration *c*_PEG_*, where *P*_DNA_ (DNA pressure in the capsid) ≤ *Π* (osmotic pressure), is insufficient to further condense DNA in the capsid. Indeed, Figure 2B demonstrates that *d_s_*-values remained constant at d_s_* ∼27.6Å between 0 and ∼36% w/w PEG8000 for wt phage λ and at d_s_* ∼29.9 Å between 0 and ∼29% w/w PEG8000 for 78% DNA phage λ. However, Figure 2B shows that when PEG concentration reached *c*_PEG_* and the osmotic pressure in solution exceeded that of DNA pressure in the capsid (*c*_PEG_ > *c*_PEG_* corresponding to *Π* > *P*_DNA_), further DNA condensation occurred, with *d_s_* gradually decreasing as PEG concentration was increased. This is a result of the ‘work’ of osmotic pressure removing water that hydrates the DNA in order to hydrate PEG polymer in solution (24).

log_10_*Π* versus *d_s_* at *c*_PEG_ > *c*_PEG_* shows a linear dependence. In order to accurately determine *c*_PEG_* value, we fit a line and extrapolate the data for log_10_*Π* versus *d_s_* where d_s_ < d_s_* to the intercept with the vertical line at constant d_s_*-value. At the intercept between two lines yields *Π* = *P*_DNA_, as shown in Figure 2B. (The observation that linear relationship between log_10_*Π* versus *d_s_* applies at d_s_ < d_s_* is discussed further in the next section.) Using this approach, we found *P*_DNA_ ∼ 34 ± 5 atm for wt phage λ and *P*_DNA_ ∼15 ± 6 atm for 78% DNA phage λ (all values are summarized in Table 1). We estimated the standard deviation in the obtained pressure values using error propagation analysis from the errors in all experimental values, described in detail in the Supplementary Materials section.

As expected, the DNA pressure in 78% DNA phage λ is lower than that in the wt phage λ, due to the lower DNA packaging density in the capsid, resulting in larger DNA–DNA separations and smaller bending stress. These intracapsid DNA pressure values are in agreement with the previously obtained (by us) DNA pressures in these phages (30) ∼25–35 atm in wt phage λ and ∼14–25 atm in 78% DNA phage λ, respectively), using the osmotic suppression assay requiring DNA ejection with help of an isolated LamB receptor (1). The accuracy of the osmotic suppression assay, however, is lower than in our new method, since it involves several biochemical treatment steps resulting in error propagation, unlike the new method. To summarize, our new ‘SAXS-osmometer’ method can be successfully applied to measure the internal genome pressure in pressurized viruses without the need to trigger DNA ejection and without requiring biochemical treatment steps. Therefore, this method can be employed as a standardizing method providing *P*_DNA_ in different viral systems.

In the next section, we determine genome pressures for a collection of different phages (including phages for which DNA pressure has not been previously obtained). By fitting the log of capsid DNA pressure (log_10_*P*_DNA_) versus *d_s_*-value in each of these viruses, we derive the equation of state for intracapsid DNA. This EOS includes contributions from both DNA–DNA interaction- and bending energy terms, unlike a previously derived EOS describing only DNA–DNA interactions in bulk DNA condensed in solution (16,23).

### The equation of state for encapsidated DNA

The SAXS-osmometer method allows us to empirically derive an EOS for encapsidated DNA, which relates capsid DNA pressure, *P*_DNA_ and *d*-spacing, *d_s_*, between tightly packaged DNA helices. To derive an EOS, we selected five different bacteriophages: P22 which belongs to the *Podoviridae* family (characterized by a short tail) as well as Utah and 9NA (both in the *Siphoviridae* family, characterized by a long noncontractile tail) and phage λ with wt DNA and phage λ with 78% of wt DNA lengths packaged in the capsid (also *Siphoviridae*) (https://ictv.global/taxonomy/) (42). These phage families encompass thousands of phage species (42). Both of these virus families package dsDNA at similar densities in a capsid with diameters of 60–70 nm (*d_s_*-values varied between ∼27 and ∼30 Å for DNA packaged in our selected phages, measured by SAXS, see Table 1), with spherical-icosahedral head geometries. This implies that the amount of bending stress on the encapsidated DNA in these phage families is similar, as genome bending is induced by a similar capsid curvature. Thus, an EOS relationship describing *P*_DNA_ versus *d_s_* for these representative phages can be applied to many dsDNA bacteriophages investigated in the literature.

**Figure 3.**
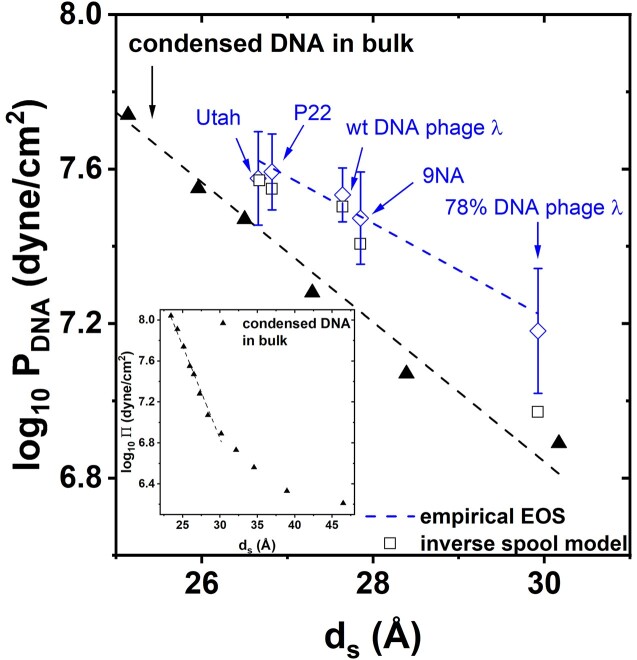
Log_10_*P*_DNA_ versus *d_s_* for all five phages in this study (blue diamonds). The fitting parameters in EOS provided by Eq. (11) are obtained from a linear fit (blue dashed line). We also show osmotic stress measurements on bulk DNA condensed in a PEG solution. SAXS-determined interaxial DNA–DNA distance, *d_s_*, was measured while gradually increasing PEG8000 concentration between 0 and 45% w/w in TM-buffer (50 mM Tris–HCl + 10 mM MgSO_4_) at 37°C (conditions identical to those of osmotic stress measurements on phage) (black triangles). The linear fit provides a semi-empirical relationship between log_10_*Π* versus *d_s_* derived by Parsegian *et al.* (48) for linear bulk DNA arrays condensed in a PEG solution, between 22 and 30 Å (black dashed line). The inset shows log_10_*Π* versus *d_s_* data for bulk DNA condensed by PEG for a wider *d_s_* range of 22–46 Å. The vertical error bars provide the uncertainty in the log of each phage pressure value obtained with the error analyses described in the Supplementary Materials section.

Analogous to the DNA pressure measurement for phage λ above, we determined pressure in phages P22, Utah and 9NA using the SAXS-osmometer method. We measured with SAXS *d_s_*-values for DNA packaged in capsids of each phage type as the osmotic pressure in the bathing solution was gradually increased through addition of PEG 8000 from 0 to 45% w/w. In Figure 2C, we show log_10_*Π* versus *d_s_* for each phage type (see raw SAXS data in Figure S1 in the Supplemental Materials). At *c*_PEG_≤ c*_PEG_, *d_s_-*values for intracapsid DNA remained constant for all phages, corresponding to d_s_*-values shown in Table 1. At *c*_PEG_> c*_PEG_, *d_s_-*values showed a linear decrease with increasing osmotic pressure on semi log-scale in Figure 2C. In order to determine the internal DNA pressure *P*_DNA_ for each phage type, we fitted a straight line to log_10_*Π* versus *d_s_* in the range of *d_s_* < d_s_*. The intercept between the linear fit for d_s_< d_s_* and the vertical line at constant d_s_* provides the DNA pressure value in the capsid, where PEG osmotic pressure, *Π*, in solution is equal to intracapsid DNA pressure, *P*_DNA_. The obtained genome pressures were: ∼39 ± 9 atm for phage P22, ∼37 ± 10 atm for phage Utah and ∼29 ± 8 atm for phage 9NA, see Figure 2C and Table 1. We estimated the standard deviation in the obtained pressure values using error propagation analysis from the errors in all experimental values, described in detail in the Supplementary Materials section. Log_10_*P*_DNA_ versus *d_s_* for all five phages (including phage λ) are plotted in Figure 3. It can be noted that the presence of ejection proteins inside phage P22 head increases overall DNA pressure in the capsid, and therefore the interaction between ejection proteins and DNA is repulsive(10). Our SAXS pressure measurement determines DNA pressure in the capsid at PEG osmotic pressure balancing the net intracapsid DNA stress, and therefore includes the contribution to the total repulsive interactions from the DNA–DNA interactions as well as the DNA-ejection proteins interactions. Furthermore, it was shown that the hydration interaction between biomacromolecules (e.g. lipids, dsDNA and proteins) displays similar repulsive force dependence on the separation between molecular surfaces <30 Å between molecule centers, where hydration interactions are not charge dependent (43,44).

Our current understanding is that unlike *Siphoviridae* virions λ, Utah and 9NA, *Podoviridae* phage P22 has so-called ‘ejection proteins’ located inside the capsid, which are ejected with the DNA during infection (10,42,45). Our previously developed osmotic suppression method (1) for DNA pressure measurement, which relies on a receptor to trigger DNA ejection, is not feasible since ‘ejection proteins’ in these phages reduce capsid volume available to DNA, contributing to higher DNA packaging density and correspondingly higher pressure. However, when DNA ejection from the capsid is triggered with receptor, these proteins are instantly released independent of PEG concentration in the outside solution *in vitro*, as it was shown for phage P22 (10). This results in an instant drop of the capsid DNA pressure, leading to an underestimate of the measured capsid pressure compared to the actual value (10). Furthermore, phages 9NA and Utah were relatively recently discovered and their cell receptors remain poorly understood (46,47). Phage Utah uses the flagellum as primary receptor, and like P22, 9NA uses surface polysaccharide as primary receptor, but their presumably required secondary receptors are not known. Our new method does not require DNA ejection, and pressure is determined in the closed capsids. This further demonstrates the robustness and broad applicability of the SAXS-osmometer method to many virus systems.

As described above, the correlation between the DNA pressure in the capsid and the *d*-spacing between packaged DNA strands, reflecting DNA concentration in the capsid, provides the equation of state for encapsidated DNA. In order to find the functional form of EOS describing our data for log_10_*P*_DNA_ versus *d_s_*, for all five bacteriophages used in this study (Figure 3), we first consider a semi-empirical relationship between *P_int_* versus *d_s_* derived by Parsegian et al (48) for linear bulk DNA arrays condensed in a PEG solution:


(1) \begin{equation*}\Pi = {P}_{int} = {F}_0{e}^{ - \frac{{{d}_s}}{c}}\end{equation*}

where *P_int_* is DNA osmotic pressure arising from DNA–DNA repulsive hydration interactions at equilibrium with PEG osmotic pressure, *Π*. This relationship was obtained from an exponential function fitting the osmotic stress measurements data (48,49), where *F*_0_ is a force amplitude and *c* is a characteristic DNA–DNA repulsion decay length. A semi-log representation was then used to simplify the experimental data analysis, by applying log_10_ to both sides of the equation to obtain a linear relationship between log_10_${P}_{int}$ and *d_s_*:


(2) \begin{equation*}{\log }_{10}{P}_{int} = - \frac{{{{\log }}_{10}e}}{c}{d}_s + {\log }_{10}{F}_0\end{equation*}

As mentioned above, this linear dependence describes only DNA–DNA interactions at *d_s_*-separations between approximately 20 and 30 Å, with dominating hydration interactions (under ionic conditions not inducing net DNA–DNA attraction, e.g. mono- and divalent salts) where decay length *c* is insensitive to cation type and to the ionic strength of the solvent medium (50,51).

Unlike free DNA condensed in a bulk solution, DNA packaged in a viral capsid experiences DNA bending stress induced by the capsid wall curvature, in addition to DNA–DNA repulsive hydration interactions (7,8,52). The intra-capsid confinement requires DNA to bend along radii that are energetically unfavorable and smaller than its persistence length, given the internal λ-capsid radius of ∼30 nm (36) and the dsDNA persistence length of 50 nm (18,7). This creates bending stress on the packaged genome. (Persistence length defines the stiffness of a polymer, describing the minimum radius of curvature it can adopt by the available thermal energy. Bending it to a smaller radius requires additional work). As mentioned above, to relieve the bending stress, DNA helices are packed closer to the capsid wall, decreasing the bending radius and also decreasing the spacing, therefore increasing the interaction energy. At the same time, the repulsive DNA–DNA interactions will push DNA strands as far from each other as possible, filling the entire capsid volume and maximizing the interstrand separations to minimize the interactions (19). Thus, there is a trade-off between bending and interaction energies balancing each other (52) and optimizing the *d_s_*-value in the capsid. Bending energy leads to capsid *d*-spacings larger than those observed in bulk solutions of DNA at the same osmotic pressure as DNA pressure in the capsid (7,8,52). Thus, both interaction energy and bending terms contribute positively to the overall DNA capsid pressure, *P_DNA_*, which can be written as:


(3) \begin{equation*}{P}_{DNA} = {P}_{int} + {P}_{bend}\end{equation*}

We rewrite this as,


(4) \begin{equation*}{P}_{DNA} = {P}_{int}\left( {1 + \frac{{{P}_{bend}}}{{{P}_{int}}}} \right)\end{equation*}

By applying log_10_ to the both sides of the equation, we obtain


(5) \begin{equation*}{\log }_{10}{P}_{DNA} = {\log }_{10}{P}_{int} + {\log }_{10}\left( {1 + \frac{{{P}_{bend}}}{{{P}_{int}}}} \right)\end{equation*}

or


(6) \begin{eqnarray*}{\log }_{10}{P}_{DNA} = - \frac{{{{\log }}_{10}e}}{c}{d}_s + {\log }_{10}{F}_0 + {\log }_{10}\left( {1 + \frac{{{P}_{bend}}}{{{P}_{int}}}} \right)\nonumber\\ \end{eqnarray*}

The obtained Eq. (6) above represents the general form of the EOS for intracapsid DNA, which includes both the interaction pressure (*P_int_*) and the bending pressure (*P_bend_*) terms. Note that the first two terms on the right side of the Eq. (6) is DNA–DNA repulsive interaction energy contribution, *P_int_*, to the total DNA pressure. The third term is associated with the additional DNA bending stress contribution, *P_bend_*, expressed relative to the DNA–DNA interaction pressure, *P_bend_/P_int_*. Both *P_int_* and *P_bend_* are functions of *d_s_* (8,23). However, it is important to stress here the observation that our empirical data for *log_10_P_DNA_* versus *d_s_* in Figure 3 displays a linear behavior. Since the DNA–DNA interaction pressure part of Eq 6, ($- \frac{{{{\log }}_{10}e}}{c}{d}_s + {\log }_{10}{F}_0)$ is a linear function, this suggests that the bending pressure part of our EOS, (${\log }_{10}( {1 + \frac{{{P}_{bend}}}{{{P}_{int}}}} )$), can also be approximated with a linear dependence, so that *log_10_P_DNA_* (*d_s_*) can be written as a sum of two lines. A mentioned above, all five phages in this study have similar capsid size with similar DNA packing densities (Table 1). This implies that while functional form of ${\log }_{10}( {1 + \frac{{{P}_{bend}}}{{{P}_{int}}}} )$ has a non-trivial behavior with *d_s_* (see discussion below), within a narrow range of *d_s_-*values, any function can be expanded as a polynomial series around an average d_s_*-value for all phages (e.g. using Taylor expansion), and described by a line:


(7) \begin{eqnarray*} && {\log }_{10}\left( {1 + \frac{{{P}_{bend}}}{{{P}_{int}}}} \right) \approx {\left[ {{{\log }}_{10}\left( {1 + \frac{{{P}_{bend}}}{{{P}_{int}}}} \right)} \right]}_{{d}_s*}\nonumber\\ && \quad +\, {\left[ {\frac{d}{{d{d}_s}}{{\log }}_{10}\left( {1 + \frac{{{P}_{bend}}}{{{P}_{int}}}} \right)} \right]}_{{d}_s*} \cdot \left( {{d}_s - {d}_{s*}} \right) + \cdots \end{eqnarray*}

Thus, our semi-empirical EOS can be written as a sum of two lines,


(8) \begin{equation*}{\log }_{10}{P}_{DNA} = - \frac{{{{\log }}_{10}e}}{c}{d}_s + {\log }_{10}{F}_0 + m({d}_s - {d}_{s*}) + A\end{equation*}

or


(9) \begin{equation*}{\log }_{10}{P}_{DNA} = - \frac{{{{\log }}_{10}e}}{c}{d}_s + {\log }_{10}{F}_0 + m{d}_s + {A}^{\prime}\end{equation*}

where A’=A-md_s_*. *m* is the slope and *A’* is the intercept with y-axis on the linear representation of the relative bending contribution to total internal DNA pressure. In order to fit our experimental data of *log_10_P_DNA_* versus *d_s_* in Figure 3 with an EOS described in Eq. (9), we first needed to determine *F*_0_ and *c* parameters describing the DNA–DNA repulsive hydration interactions alone, without bending term contribution. *F*_0_ and *c* parameters describe universal DNA–DNA interaction behavior both in free DNA condensed in a bulk solution (Eq. 2) and for DNA packaged in a viral capsid (Eq. 9), when in both cases, the repulsion is dominated by hydration interaction at *d_s_* ∼20–30 Å (16,23). Therefore, we independently performed osmotic stress measurements on bulk DNA condensed in a PEG solution. We measured with SAXS the interaxial DNA–DNA distance, *d_s_*, while gradually increasing PEG8000 concentration between 0 and 45% w/w in TM-buffer (50 mM Tris–HCl + 10 mM MgSO_4_) at 37°C (conditions identical to those of osmotic stress measurements on phage), see Figure 3. We used Eq. (2) to fit a linear relationship describing log_10_*Π* versus *d_s_* between 22 and 30 Å (black dashed line), where observed linear regime corresponds to dominant DNA–DNA hydration interaction (16,23,53). We obtained:


(10) \begin{equation*}lo{g}_{10}{\mathrm{\Pi }} = lo{g}_{10}{P}_{int} = - 0.1809{d}_s + 12.27\end{equation*}

The fit yields *F*_0_ = 1.8607 × 10^12^ dyn/cm^2^ and *c*-value of 0.24 nm, which is within the previously reported range for *c* ∼0.2–0.3 nm describing decay length of DNA–DNA interactions dominated by repulsive hydration forces (48,50,51). Note that at DNA–DNA separations >30 Å, the electrostatic interactions become dominant over hydration interactions and log_10_*Π* values start to deviate from a linear regime (with a possible structural transition from a long-ranged hexagonal order to a cholesteric order) (54), see the inset in Figure 3 showing log_10_*Π* versus *d_s_* data for bulk DNA in *d_s_* range 22–46 Å. It is interesting to observe that *d_s_-*values continuously decrease as *Π* is gradually increasing for bulk DNA in PEG8000 solution, since DNA osmotic pressure is at equilibrium with PEG osmotic pressure at all PEG concentrations. However, for DNA packaged in a phage capsid, *d_s_* is constant at PEG concentrations *< c*_PEG_* and is only decreasing above *c*_PEG_* PEG concentration. This is explained by the fact that DNA pressure in the capsid is not at equilibrium with osmotic pressure of the bathing solution until *Π = P*_DNA_ is reached, since DNA is initially confined mechanically by the capsid walls, independent of the bathing solution conditions.

Finally, we can now fit EOS in Eq. (9), using


(11) \begin{equation*}{\log }_{10}{P}_{DNA} = - 0.1809{d}_s + 12.27 + m{d}_s + {A}^{\prime}\end{equation*}

as a function of *d_s_* to describe our data in Figure 3. The obtained linear fit, shown with the dashed blue line in Figure 3, has a slope *m =*0.0596 Å^−1^ and an intercept with *y*-axis $A^{\prime} = - 1.413$ dyne/cm^2^ (note that the slope *m* is positive, which gives rise to a ‘less negative’ slope for *log_10_ P_DNA_* in comparison to the slope describing bulk DNA condensation, log_10_*P*_int_versus *d_s_*, black dashed line in Figure 3). As seen in the Figure, the EOS expressed as a sum on two lines (one describing DNA–DNA interaction and one describing DNA bending pressure), provides a good description of our data and can be used to predict DNA pressure in spherical-icosahedral viral capsids with diameters 60–70 nm, where bending stress is similar. Based on the error analysis of all experimental values used for the pressure value determination, described in the Supplementary Materials section, our empirical EOS for the set of studied bacteriophages has an uncertainty varying between ± 1.7 atm and ±4.6 atm for the range of *d_s_* values corresponding to DNA packing density in phage systems studied in this work (providing the minimum pressure detection level). [The EOS measured above describes DNA behavior in bacteriophages with spherical-icosahedral capsids within the investigated size range at high DNA packing densities, where DNA–DNA interactions are dominated by hydration forces, and at given ionic conditions and temperature. We anticipate that bending energy term dependence on *d_s_* will be affected by a significant change in capsid size, providing different curvature. However, as mentioned above, the range of phages studied here provides parameters for EOS applicable to a majority of phage systems, due to similar dimensions and geometry. Also, using our pressure measurement method, we plan to expand our empirical EOS analysis to encompass other viral systems with different dimensions, e.g. Herpesvirus family with capsid diameters of ∼120–200 nm but with similar DNA packing densities]. The empirically derived EOS with the fitted parameters *m* and *A’* allow us to calculate *P_bend_/P_int_* ratio for each phage system (at each *d_s_*-value) in our study, using the relationship ${\log }_{10}( {1 + \frac{{{P}_{bend}}}{{{P}_{int}}}} ) = m{d}_s + A^{\prime}$. As limit cases, for the largest interstrand distance of *d_s_*= 29.92 Å in 78% DNA phage λ (lowest phage DNA packing density in our study), we obtain *P_bend_/P_int_* ≈ 134%, suggesting that bending stress dominates over the interaction energy contribution to the total DNA capsid pressure. On the contrary, at the smallest interstrand distance of *d_s_*= 26.67 Å in phage Utah (highest phage DNA packing density in our study), the predicted *P_bend_/P_int_* ≈ 50%. This suggests that the interaction energy dominates the DNA pressure in the capsid. As seen in Figure 3, as *d_s_*-values are decreasing, the blue dashed line describing the DNA pressure in phage capsids is converging with the black dashed line, which describes pure repulsive hydration interaction in bulk DNA. This observation makes sense since *P_int_* is a faster growing function of *d_s_* than *P_bend_* (8). We can estimate *P_int_* alone at each given *d_s_*-value from the linear fit to bulk DNA data (dashed black line in Figure 3) and then calculate *P_bend_* contribution using *P_bend_/P_int_* calculations above. Indeed, for 78% DNA phage λ we obtained *P_int_* ∼ 6.4 atm and *P_bend_* ∼ 8.6 atm. For phage Utah, we obtained *P_int_* ∼ 24.7 atm and *P_bend_* ∼ 12.4 atm. Thus, in the limit of small *d_s_*-values, we expect phage DNA to behave similarly to bulk DNA with DNA capsid pressure approaching the osmotic pressure of DNA condensed in solution; that is, Eq. (6) converges to Eq. (4), when *P_int_ >> P_bend_*. It should be mentioned that the entropy change contribution from DNA confinement in the capsid is included in the EOS above. Specifically, it has been previously shown that the empirical relation describing hydration interaction between hexagonally ordered DNA strands (derived with the osmotic stress measurements on bulk DNA), corresponding to the first two terms in the Eq. (11) above, contains hydration entropy term. More precisely, as DNA becomes hexagonally packed, the ordered water molecules directly surrounding the DNA are released, increasing the net disorder of the system, thus increasing its entropy. The positive change in entropy associated with the dehydration process upon DNA packing has been observed by Leikin *et al.* in dense hexagonal phases of DNA probed by the osmotic stress method (28). In their study, they were able to convert osmotic stress data into changes in entropy and enthalpy per base pair. The change in entropy associated with DNA packing induced by osmotic pressure was positive and dominated the negative DNA conformational entropy loss. We confirmed this independently for DNA packaged in phage λ capsids, using isothermal titration calorimetry to measure enthalpy change of DNA ejection from capsids and deriving the entropy change term from the free energy calculation (29).

In light of these findings of experimentally derived bending stress contribution to the DNA pressure in a viral capsid, it is interesting to revisit the previously proposed and commonly used theoretical model prediction of DNA bending stress contribution based on the inverse spool model of DNA packaged in a spherical capsid with a cylindrical void in the capsid center (7,55) (the void is generated due to the bending stress pushing the DNA out from the center of the capsid volume, in order to minimize its curvature). The analytical expression for bending energy *E_bend_*, in ref (55) is expressed in terms of temperature *T*, interstrand distance *d_s_*, DNA persistence length *l_p_*, inner capsid radius *R_out_*, and radius of the cylindrical void *R*:


(12) \begin{eqnarray*} && {E}_{bend}\left( R \right) = - \frac{{4\pi {l}_p{k}_BT}}{{\sqrt 3 d_s^2}}\nonumber\\ && \quad \left[ {\sqrt {R_{out}^2 - {R}^2} + {R}_{out}\ln \left( {\frac{{{R}_{out - }\sqrt {R_{out}^2 - {R}^2} }}{R}} \right)} \right]\end{eqnarray*}

This bending energy expression can be used to estimate *P_bend_* via the thermodynamic relationship ${P}_{bend} = - \frac{{\partial {E}_{bend}}}{{\partial {V}_c}}$, where *V_c_* is the volume of the capsid. Thus, to calculate *P_bend_* we need to apply the chain rule,


(13) \begin{equation*}{P}_{bend} = - \frac{{\partial {E}_{bend}}}{{\partial {R}_{out}}} \cdot \frac{{\partial {R}_{out}}}{{\partial {V}_c}}\end{equation*}

If ${V}_c = \frac{{4\pi R_{out}^3}}{3}$ then,


(14) \begin{equation*}{P}_{bend} = - \frac{1}{{4\pi R_{out}^2}} \cdot \frac{{\partial {E}_{bend}}}{{\partial R}} \cdot \frac{{\partial R}}{{\partial {R}_{out}}}\end{equation*}

In the inverse spool model, *R* and *R_out_* are related through


(15) \begin{equation*}R = \sqrt {R_{out}^2 - {{\left( {\frac{{3\sqrt 3 d_s^2L}}{{8\pi }}} \right)}}^{\frac{2}{3}}} \end{equation*}

where *L* is the length of viral DNA packaged in a capsid. After solving the derivatives and with algebraic simplification, we obtain


(16) \begin{equation*}{P}_{bend} = \frac{{{l}_p{k}_BT}}{{\sqrt 3 d_s^2{R}_{out}}} \cdot \frac{{\sqrt {R_{out}^2 - {R}^2} }}{{{R}^2}}\end{equation*}

For all DNA-filled phage capsids in our study, *R/R_out_* ≪ *1*, that is, inner capsid radius is much larger than the calculated radius of the cylindrical void. Hence, we can apply a series expansion to the square root term of the equation and simplify it to


(17) \begin{equation*}{P}_{bend} \approx \frac{{{l}_p{k}_BT}}{{\sqrt 3 d_s^2{R}^2}}\end{equation*}

which is the same expression as previously reported by Odijk (56) for DNA bending stress inside a phage capsid using a mesoscopic scale continuum computation model for *R/R_out_* ≪ *1* limit case.

We can now calculate the third term in our EOS expression in Eq 6 by substituting the analytical expressions for *P_ben_*_d_ from Eq. (17) and *P_int_* from Eq. (1), so that ${\log }_{10}( {1 + \frac{{{P}_{bend}}}{{{P}_{int}}}} ) = {\log }_{10}( {1 + \frac{{\frac{{{l}_p{k}_BT}}{{\sqrt 3 d_s^2{R}^2}}}}{{{F}_0{e}^{ - \frac{{{d}_s}}{c}}}}} )$. Using *F*_0_ and *c* parameters that were empirically obtained above from the osmotic stress measurements on bulk DNA condensed in PEG solution (Figure 3), we can provide a theoretical prediction for log_10_*P*_DNA_ versus *d_s_* for the inverse spool model of encapsidated DNA, which includes both repulsive interactions and bending stress contributions to the total internal DNA pressure for each phage type in our study, shown as black empty squares in Figure 3 (inner capsid radii used for calculation are shown in Table 1). It is interesting to observe that the theoretical model prediction describes adequately well log_10_*P*_DNA_ values for phages with higher DNA packing densities (smaller *d_s_* values), in phages wt phage λ, P22, 9NA and Utah. However, for the lower DNA packing density in 78% DNA phage λ, the agreement is poor with the inverse spool model underestimating the bending pressure contribution to the total DNA pressure. As discussed above, our data in Figure 3 shows that at higher packing densities (small *d_s_*), the DNA–DNA interaction pressure contribution, *P_int_*, dominates the DNA pressure in the capsid and *P_bend_* is relatively small, while at low packing densities *P_bend_* becomes the major contribution to the total DNA pressure. That implies that the inverse spool model underestimates the bending pressure of DNA packaged in viral capsids in general but the discrepancy becomes more problematic for viruses with lower DNA density where bending stress dominates the total DNA pressure. Indeed, while the inverse spool model assumes an empty void in the center of the capsid, we have observed with cryo-EM reconstruction (19) that packaged DNA is in fact dispersed throughout the entire λ-capsid volume without a void. This could potentially explain why the theoretical model underestimates the bending stress of intracapsid DNA.

## Concluding remarks

We established a novel SAXS-based method that allows for measurement of DNA pressure in viral capsids. This method does not require triggering DNA ejection from the capsids with a receptor isolated from cells, unlike the previously used assay (1). This allows determination of capsid pressures in many viral systems for which receptors are either not known or cannot be successfully isolated. Furthermore, this pressure measuring approach avoids interference from a specific genome ejection mechanism that can affect the extent of DNA ejection during its equilibration with the external osmotic pressure (e.g. presence of ejection proteins in the capsid (10)).

We mapped DNA capsid pressures for a collection of phages with similar capsid dimensions and therefore comparable DNA bending stress imposed by the curvature of the capsid wall (range of capsid diameters 63–68 Å). Our data shows a linear relationship between the log of DNA capsid pressure (log_10_*P*_DNA_) and interstrand distance, *d_s_*. Using a general expression form for DNA pressure in the capsid, written as a sum of DNA–DNA interaction and DNA bending stress contributions to the total pressure, we were able to derive an empirical equation of state, which describes well our data of *log_10_P_DNA_* versus *d_s_*. The resulting EOS is a sum of two lines on a semi-log scale, where one line is the previously found interstrand interaction pressure (log_10_*P*_int_ versus *d_s_*) (14,16) and the second line describes the bending stress pressure (log_10_*P*_bend_ versus *d_s_*). It is the first time that DNA bending stress in a capsid could be measured separately from DNA–DNA interaction. It is striking to observe that the line describing the DNA pressure in a capsid, log_10_*P*_DNA_(*d_s_*) converges at small *d_s_*-values with the line describing DNA interaction pressure alone measured separately for free DNA condensed by PEG in a bulk solution, log_10_*Π*(*d_s_*). This suggests that DNA–DNA repulsive hydration interaction dominates the intracapsid genome pressure for viruses with high packing densities. This explains a previous observation where DNA capsid pressure in fully packaged wt phage λ could be approximated with an empirical expression for DNA osmotic pressure in the bulk (1) (14,16,23). On the contrary, for viruses with larger *d_s_*, bending stress dominates the pressure in the capsid and its contribution alone is on the order of ∼10 atmospheres or higher. This suggests that, in viruses with lower DNA packing densities, DNA bending stress generates a sufficient amount of force that could be responsible for gene release during infection. Indeed, for clinically highly relevant viruses such as HBV (57) (packaged with partial dsDNA) and AAV (58) (packaged with self-complementary DNA), it has been observed that genomes are packaged under stress despite packing densities being significantly lower than in dsDNA phages (capsids of these viruses have diameters of ∼30 nm, compared to ∼65 nm for phages in this work, which implies that genome bending stress is even higher than in phage). Our SAXS-osmometer method can be used to determine the actual DNA pressure in these viruses, while the empirically obtained EOS provides a long-needed prediction of the magnitude of capsid genome pressure in many viral systems with similar dimensions and capsid geometry. Also, because the inner capsid wall of lambdoid phages is negatively charged (19,36), there is no attractive interaction between the DNA and the inner capsid surface. The repulsive interaction between the outermost DNA layer and the capsid wall contributes positively to the pressure, which is reflected in our pressure measurement as well as in the empirically derived EOS.

These findings will facilitate the discovery of mechanisms for genome uncoating and encapsidation during viral infectious cycle and in parallel advance the knowledge of physical chemistry of sterically confined DNA condensates. Furthermore, this work could find application in the design of new antivirals (using multivalent cationic compounds that reduce DNA pressure and block DNA ejection (9)), as well as the design of gene therapy vectors assembled under controlled thermodynamics conditions in order to efficiently package non-viral DNA vectors (e.g. AAV-based gene therapy) (58).

## Supplementary Material

gkad852_Supplemental_File

## Data Availability

The data underlying this article are available in the article and in its online supplementary material.
