# Supplementary material for: ‘SAXS-osmometer’ method provides measurement of DNA pressure in viral capsids and delivers an empirical equation of state
Source: Nucleic Acids Res. 2023 Oct 27;51(21):11415–27. doi: 10.1093/nar/gkad852 (PMC10681747; doi:10.1093/nar/gkad852)
Supplement: gkad852_Supplemental_File [file gkad852_supplemental_file.docx]

Supplemental materials

**‘SAXS-osmometer’ method provides direct measurement of DNA pressure in viral capsids and delivers an empirical equation of state**

José Ramon Villanueva Valencia^1^, Dong Li^2^, Sherwood R. Casjens^3^, and Alex Evilevitch^1,2*^

*^1^Department of Experimental Medical Science and NanoLund, Lund University, Box 124, Lund, Sweden*

*^2^Physics Department, Carnegie Mellon University, Pittsburgh, PA, 15213, USA*

*^3^Division of Microbiology and Immunology, Department of Pathology, University of Utah School of Medicine, Salt Lake City, Utah, 84112, USA*

** Corresponding author e-mail: Alex.Evilevitch@med.lu.se*

| 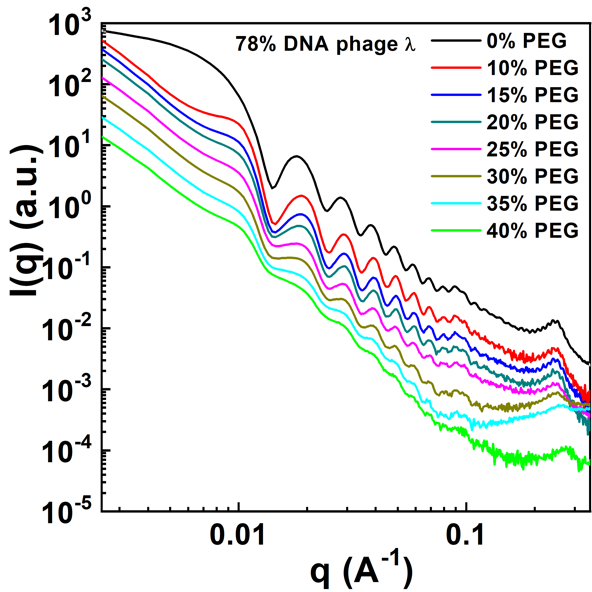 | 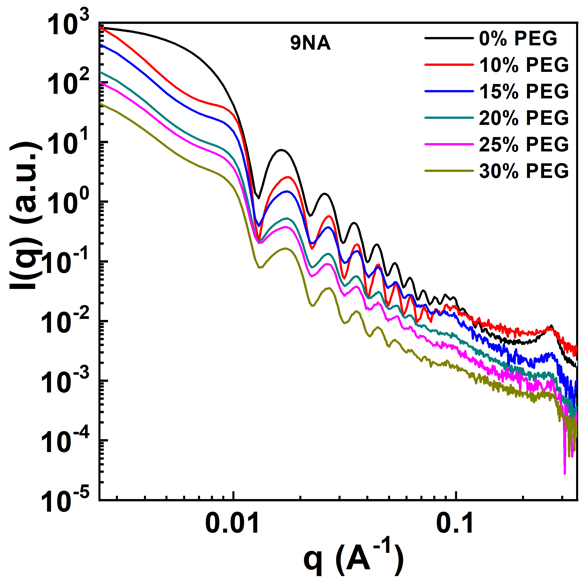 |
| --- | --- |
| 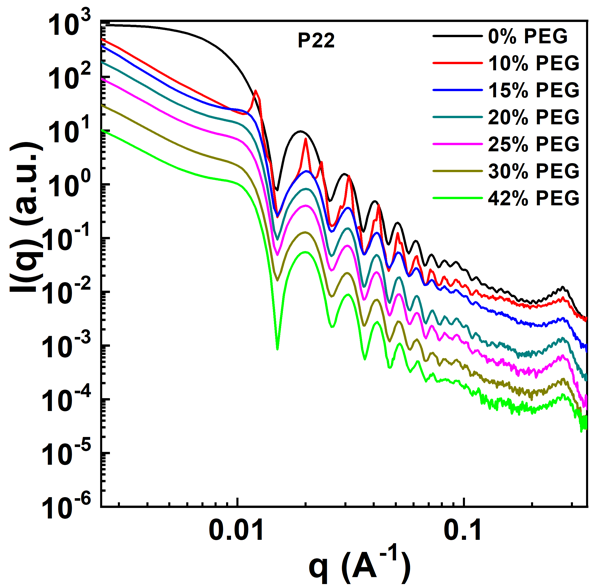 | 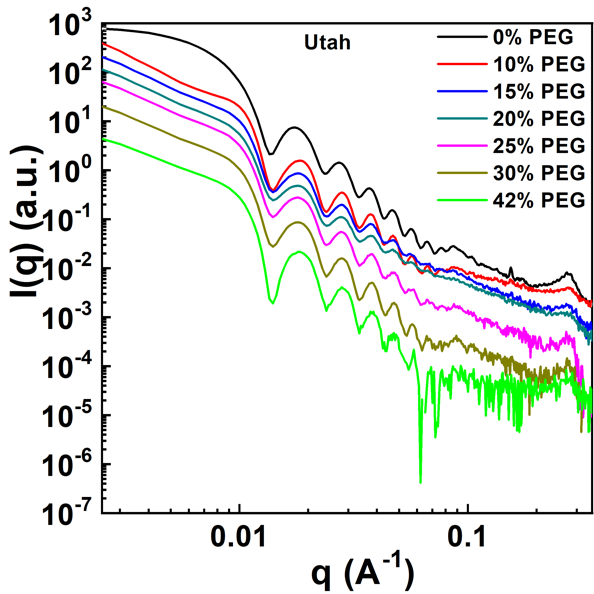 |

**Figure S1.** SAXS intensity profiles, *I(q)* versus PEG concentration for phages: 78% DNA phage λ, 9NA, P22 and Utah in Tris-MgSO_4_ buffer at 37°C.

**Calculation of the standard deviation in the pressure measurement method and in the EOS**

Here, we summarize the step-by-step error analysis used to determine the standard deviation in the measured capsid pressure values in the studied phage systems as well as in the equation of state (EOS). The standard deviation for each pressure measurement obtained with the error propagation analysis is shown in Table 1 (as well as error bars in Figure 3). The average estimated uncertainty in the pressure values predicted by the EOS varies between ±1.7 atm and ±4.6 atm for the range of DNA-DNA spacing values (*d_s_*) for bacteriophages in this study.

*Error in the prepared concentration of the PEG solutions and the corresponding osmotic pressure:*

Due to difficulty of pipetting the viscous PEG solutions, we tried to minimize systematic and statistical errors that occur when the solution adheres to the pipette tips. A stock solution of 50% by weight (w/w) PEG 8000 was prepared in Tris-MgSO_4_ (TM-buffer). This stock solution was used to prepare solutions of PEG/TM-buffer at various specified % (w/w) on an analytical balance within an error in concentration of approximately ~0.1% (w/w). Then, we applied error propagation analysis to estimate the error in the osmotic pressure values obtained with the empirical relationship from ref.[1] to convert the PEG 8000 % (w/w) to the osmotic pressure, using *Π(atm) = -1.29 G^2^T + 140G^2^ + 4G,* where *G = w/(100-w)*, with *w* being the weight percent (% w/w). The obtained uncertainty in the osmotic pressure values (*Π*) varies with PEG concentration and is between ~1% and ~5% atm. On the semi-log representation of *log_10_Π* versus *d_s_* in Figure 2, however, these vertical error bars are smaller than the symbols shown in the figure. Nevertheless, the error in the PEG concentration was included in the estimate of the errors in the obtained DNA pressure for each phage and in the EOS, as described below.

*Error in the DNA-DNA interstrand distance determined by SAXS:*

Figure 2A exemplifies how the “broad peak” model was applied to determine the location of the Bragg’s peak maximum for ordered DNA in phage capsid at each % w/w PEG. This maximum value of the peak is converted to the interstrand distance for a hexagonal lattice array, $d_{s}=\frac{4\pi}{\sqrt{3}q_{Bragg}}$. The location of the peak is determined within one standard deviation error, σ_qBragg_, where *q*_Bragg_ is the peak center. The standard deviation was calculated with *σ_ds_ = d_s_σ_qBragg_/q_Bragg_*, shown as horizontal error bars in Figures 2B and 2C (see also Table 1).

*Error in the pressure values determined with SAXS-osmometer method:*

As described in the main text, we use a line to fit *log_10_Π* versus *d_s_* at C_PEG_ > C^*^_PEG_ in Figures 2B and C, where DNA in the capsid is becoming gradually condensed with increasing PEG concentration. Determination of the errors in the fitting line to the semi-log plot of *log_10_Π* versus *d_s_* in Figures 2B and C (the sloped line at C_PEG_ > C^*^_PEG_) is based on the *weighted least-squared method* described in ref. [2]. This method takes into account the errors in both variables: *log_10_Π* and *d_s_*. If (*X_i_,Y_i_*) and *(σ_x,i_,σ_x,i_*) are the experimentally observed data points and their errors (*i=*1,2,3,..), respectively, the best line *y=a+bx* that fits the experimental data is described by the following parameters:

$$a=\bar{Y}-b\bar{X}$$

$$b=\frac{\sum W_{i}\beta_{i}V_{i}}{\sum W_{i}\beta_{i}U_{i}}$$

$$\sigma_{a}^{2}=\frac{1}{\sum W_{i}}+\bar{x}^{2}\sigma_{b}^{2}$$

$$\sigma_{b}^{2}=\frac{1}{\sum W_{i}u_{i}^{2}}$$

The slope of the line, *b*, and the intercept with *y*-axis, *a*, and their respective standard deviations, *σ_a_* and *σ_b_*, are written as variables, which are described in ref. [2].

The slope, *b*, and the intercept to *y*-axis, *a*, of the sloped line fitting *log_10_Π* versus *d_s_* at C_PEG_ > C^*^_PEG_ are used to extrapolate the line to the average interstrand distance value, *d_s_**, described by the vertical line in Figures 2B and C (at C_PEG_ ≤ C*_PEG_). The intercept value between the sloped- and vertical lines provides the PEG osmotic pressure value (C*_PEG_) equal to that of the DNA pressure in the capsid, *P_DNA_*. We determine the uncertainty $\sigma_{z}$ in *P_DNA_* value by using the expression below, where *z = log_10_Π*, see the details of this error propagation analysis in ref. [3]:

$$\sigma_{z}=\sqrt{{(bd_{s}^{*})}^{2}\left[ \left( \frac{\sigma_{b}}{b} \right)^{2}+\left( \frac{\sigma_{ds*}}{d_{s}^{*}} \right)^{2} \right]+\sigma_{a}^{2}}$$

This expression calculates the uncertainty in the determination of the viral internal DNA pressure provided by the intercept between the vertical line at constant *d_s_** (at C_PEG_ ≤ C*_PEG_) and the sloped line (at C_PEG_ > C*_PEG_), as shown in Figures 2B and 2C. The interstrand distance *d_s_** (provided by the vertical line before PEG-induced DNA condensation) and its associated standard deviation *σ_ds*_*, and the sloped line at higher % PEG (where *d_s_ < d_s_**) with the slope *b*, the intercept with the y-axis *a* and the associated standard deviations *σ_a_* and *σ_b_*, respectively, are the variables required to calculate the uncertainty in the viral internal pressure. The resulting standard deviation $\sigma_{z}$ in *P_DNA_* is shown in Figure 3 (vertical error bars) for the collection of phages in the semi-log representation of the experimental data. The conversion from *σ_z_* (log scale) to *σ_Π_* (linear scale) is achieved through the relationship *σ_Π_=P_DNA_σ_z_ ln10*, where *P_DNA_* is the internal capsid DNA pressure. Error *σ_Π_* is shown next to the pressure value for each phage in Table 1.

*Error in the EOS*

In order to estimate the accuracy of the proposed EOS, we apply the same weighted least-squared method as described in ref. [2] to the experimental points log_10_P_DNA_ versus *d_s_* shown in Figure 3 (blue diamonds). We can determine the best parameters for the slope, *b*, and the intercept with *y*-axis, *a*, that describe the EOS, as well as the standard deviation $\sigma_{z}$ (including the errors in both horizontal- and vertical axis). The standard deviation $\sigma_{z}$in the EOS (where *z = log_10_Π*), is provided by the following expression:

$$\sigma_{z}=\sqrt{\frac{1}{N-2}\sum_{i=1}^{N} \left( z_{i}-a-bd_{s,i} \right)^{2}}$$

Geometrically, this expression calculates the deviation of the weighted least-squared-adjusted data points from the experimentally observed data points. *N* is the number of experimental data points, *z_i_* is log_10_ of the osmotic pressure value at each point and *d_s,i_* is the corresponding DNA interstrand distance. The resulting standard deviation in *P_DNA_* is shown in Figure 3 as vertical error bars for the collection of phages studied in our work. The obtained uncertainty in the fitted line in Figure 3, describing our EOS, is *σ_z_*=0.05124 (on a log scale of dyne/cm^2^). The conversion from *σ_z_* (log scale) to *σ_Π_* (linear scale) is provided by the relationship *σ_Π_=P_DNA_σ_z_ ln10*, where *P_DNA_* is the internal capsid DNA pressure. The obtained standard deviations vary between ±1.7 atm and ±4.6 atm for the range of *d_s_* values corresponding to DNA packing density in phage systems studied in this work.

**References**

1. Parsegian, V.A., et al., *Osmotic-Stress for the Direct Measurement of Intermolecular Forces.* Methods in Enzymology, 1986. **127**: p. 400-416.

2. York, D., et al., *Unified equations for the slope, intercept, and standard errors of the best straight line.* American journal of physics, 2004. **72**(3): p. 367-375.

3. Taylor, J.R. and W. Thompson, *An introduction to error analysis: the study of uncertainties in physical measurements*. Vol. 2. 1982: Springer.
